# Intrahepatic Biliary Cystadenoma With Intracystic Gallstone Formation

**DOI:** 10.1155/1994/62393

**Published:** 1994

**Authors:** Shize Lei, Don R. Domenico, John M. Howard

**Affiliations:** 1 Department of Pathology Toledo Hospital and Medical College of Ohio Toledo Ohio USA; 2 Department of Pathology Toledo Hospital Toledo Ohio USA; 3 Department of Surgery Mercy Hospital Medical College of Ohio Toledo Ohio USA; 4 Harris McIntosh Tower 2121 Hughes Drive Suite 940 Toledo OH 43606 USA

## Abstract

Biliary cystadenoma is a rare tumor of the liver. We describe a biliary cystadenoma of the left lobe of the liver with intracystic gallstone formation. This is the first report of stone formation in biliary cystadenoma of the liver.


*HPB Surgery*, 1994, Vol. 7, pp. 241-249
Reprints available directly from the publisher
Photocopying permitted by license only

© 1994 Harwood Academic Publishers GmbH
Printed in the United States of America

### CASE REPORT

### INTRAHEPATIC BILIARY CYSTADENOMA WITH INTRACYSTIC GALLSTONE FORMATION

SHIZE LEI
*Department of Pathology, Toledo Hospital and Medical College of Ohio, Toledo, Ohio, USA*

DON R. DOMENICO
*Department of Pathology, Toledo Hospital, Toledo, Ohio, USA*

JOHN M. HOWARD
*Department of Surgery, Mercy Hospital, Professor: Medical College of Ohio, Toledo, Ohio, USA*

*(Received 15 December 1992)*

Biliary cystadenoma is a rare tumor of the liver. We describe a biliary cystadenoma of the left lobe of the liver with intracystic gallstone formation. This is the first report of stone formation in biliary cystadenoma of the liver.

KEY WORDS: Biliary cystadenoma, intracystic gallstones

Biliary cystadenomas are rare tumors which have a malignant potential. They are usually located in the liver, less frequently in the bile ducts. Most published cases have been single case reports[1-6], the largest series being reported by Wheeler and Edmondson[7]. We present the first case occurring with gallstone formation within the cystadenoma.

### CASE REPORT

A 45-year-old white woman was hospitalized in September 1991 for gastric bypass for morbid obesity. She had been obese for many years and had undergone a cholecystectomy for gallstones seven years earlier. No stones had been noted in the bile ducts.She was asymptomatic relative to the gastrointestinal tract. An upper

Address correspondence to: John M. Howard, M.D., Harris McIntosh Tower, 2121 Hughes Drive, Suite 940, Toledo, OH 43606, USA

gastrointestinal series was normal. Her total bilirubin was 0.5 mg%; glucose, 158 mg%; cholesterol, 158 mg%; triglyceride, 86 mg%; alkaline phosphatase, 57 Bodansky units; serum glutamic oxaloacetic transaminase (SGOT), 23 units.

Laparotomy revealed a huge cystic mass filling the left lobe of the liver. Intraoperative ultrasound demonstrated a large multilocular cyst. The remainder of the liver was normal in appearance as were the kidneys and pancreas. Dynamic Doppler studies demonstrated a large collection of static fluid within the cyst, displacing the hepatic arteries and veins. There was no sonographic evidence of large vascular connections to the cystic tumor nor was there evidence of any abnormality, including stones, of the extrahepatic bile ducts. The decision was made to close the abdomen without gastric bypass in order to gain further information and to discuss the findings with the patient. On a subsequent contrast-enhanced CT scan, the cyst measured 14 × 11 cm. It was surrounded by a thin but well defined capsule and appeared to be septated (Figure 1). Angiography revealed the mass to be relatively avascular.

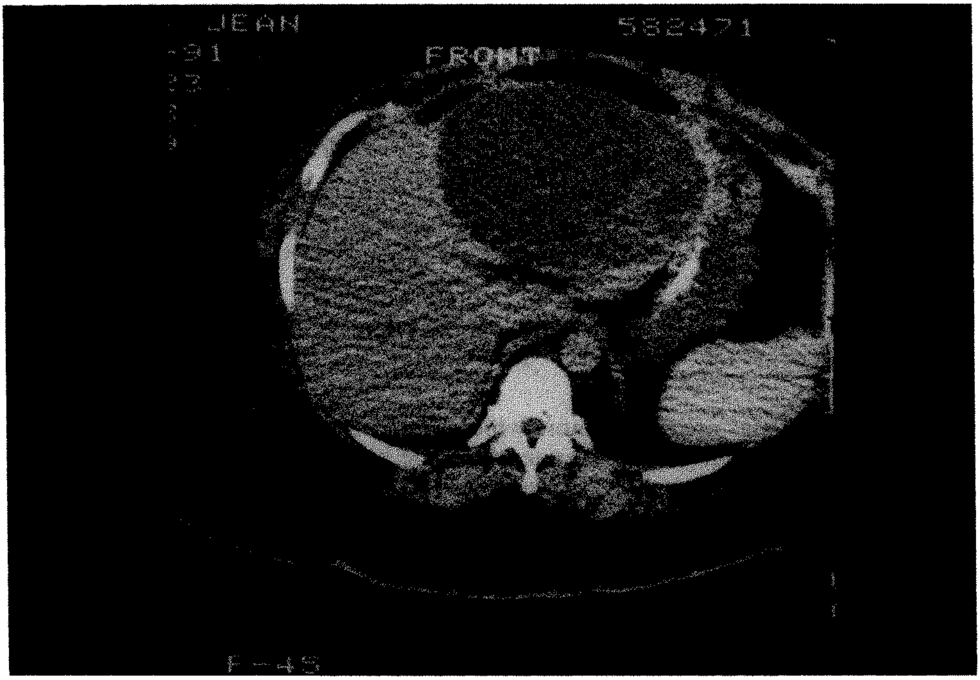

**Figure 1** Computed tomographic scan. A multiseptated cystic mass is seen replacing the left lobe of the liver.

Secondary operation was performed four days after the first laparotomy. The cyst was found to largely replace the left lobe of the liver (Figure 2). The common bile duct, portal vein and the right and left hepatic arteries and their proximal

**Figure 2** A huge glocular mass with smooth surface in the left lobe of the liver. (*See colour plate I at the back of this issue*)

branches were isolated. Most of the left lobe of the liver, including the cystic mass was then resected, guided in part by intraoperative ultrasound. Convalescence was uneventful, the patient being discharged 14 days later. She has remained well over the ensuing 10 months.

On gross examination, the cystic tumor was globular with a smooth external surface. Its diameters measured 14 × 12 × 8 centimeters. On cut surface, the tumor was multicystic; some cysts containing dark greenish bile-like fluid and some clear serous fluid, containing crystalline materials. On opening every cystic space, there was a large dilated duct-like structure, connecting to a central cyst, which had a lining very similar to the mucosa of the gallbladder. This latter structure was thought perhaps to be an aberrant bile duct in direct communication with the cystadenoma because bile refluxed from it when it was divided. Multiple darkyellow stones, measuring 0.2–0.7 cm in diameter were seen in this aberrant biliary structure. The multicystic spaces were lined by a smooth and glistering surface (Figure 3).

Histological examination revealed the cysts to be lined by a layer of columnar mucin-secreting cells. The nuclei were of uniform size and had fine chromatin. No mitotic figures were observed. A few polyp-like structures were found to have cores of varying vascularity. The subepithelial stroma consisted of densely arranged spindle cells. In areas there were mucosal ulcerations with bile pigment. A few lymphocytes and neutrophils were observed in the stoma (Figure 4). No evidence of malignancy was seen on extensive sampling. The diagnosis of biliary cystadenoma with mesenchymal stroma (CMS) of left lobe of the liver was made.

**Figure 3** The cystadenoma, opened to demonstrate a biliary type mucosa, bile-like fluid (arrow) and stones (arrowhead). (*See colour Plate II at the back of this issue)*

### DISCUSSION

Multilocular biliary cystadenomas of the liver are rare in comparison to unilocular cysts. Fewer than 100 cases of biliary cystadenomas have been reported. To more adequately differentiate tumors of this type, Wheeler and Edmondson[7] stressed the importance of the stroma component, referring to their tumor as "biliary cystadenoma with mesenchymal stroma (CMS)", they found that only 27 reported cases appeared to meet the diagnostic criteria of CMS in the English literature. Their

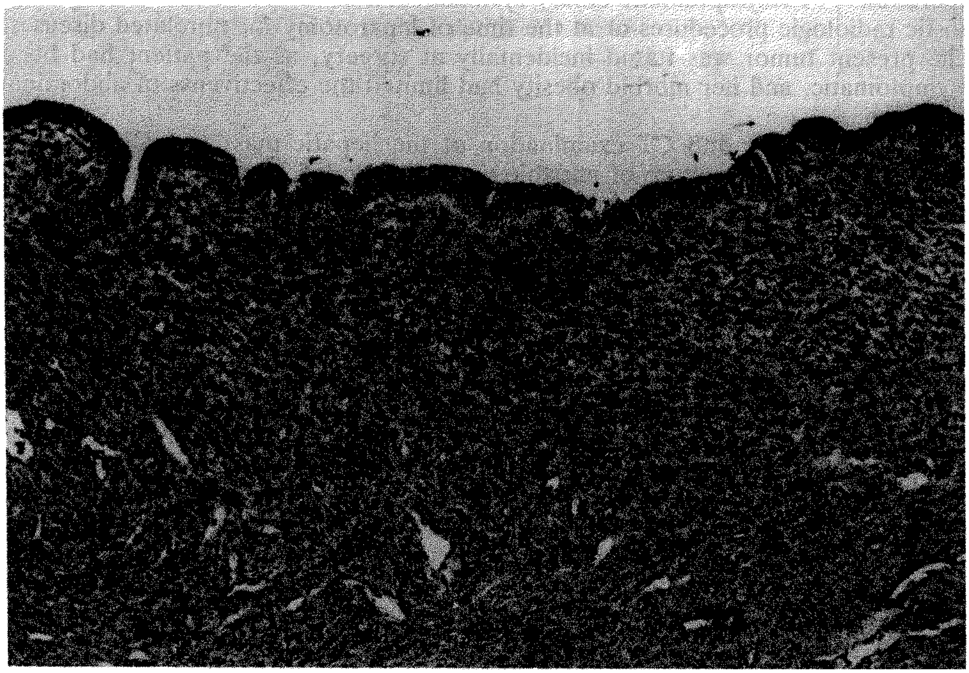

**Figure 4** Cystadenoma showing mucinous epithelium and underlying compact stroma. (Magnification × 200, stain H & E)

criteria for CMS have been generally accepted by pathologists[8]. We have been unable to find a previous example of a cystadenoma occurring after cholecystectomy with stone formation within the cyst.

Biliary cystadenomas are characterized histologically by cuboid or columnar cells, lining a serous or mucinous filled multiseptated cavity, typically with papillary infolding and focal thickening of definite stroma. In their report, Wheeler and Edmondson described the hepatobiliary cystadenoma with mesenchymal stroma, as a homogenous clinicopathologic group, occurring exclusively in women[7]. They concluded that cystadenomas without mesenchyma stroma occurred more frequently in men, and constituted a significantly different lesion. The present tumor meets the criteria of the biliary cystadenoma with mesenchymal stroma (CMS).

Almost all the patients reported with classic cystadenoma with mesenchymal stroma (CMS), including the present one, were female[1,2,5–7]. The patients are usually in middle age[7] at the time of diagnosis. Only three children have been reported[12,13], including a recent report of a 3-year old boy whose tumor was without mesenchymal stroma[13]. Simpler unilocular solitary cysts, however, are more frequent in children[14].

The symptoms and signs vary. The majority of patients with hepatic biliary cystadenoma present with abdominal swelling and/or a palpable abdomen mass. Abdominal pain has been noted in over half of the patients[7,11,16]. Jaundice may rarely be the chief complaint, secondary to biliary obstruction as we and others have previously observed [9,17]. A few cases have been diagnosed prior to operation[7,11,16]. Asymptomatic biliary cystadenomas have been found during diagnostic radiologic procedures or at the time of laparotomy for unrelated diseases. The present tumor was found incidentally at surgery, as the patient had been asymptomatic, and her morbid obesity had limited the effectivenss of abdominal palpation.

Ultrasonographic and CT examination of the hepatic mass typically shows a multilocular cystic structure of variable size, surrounded by normal parenchyma. Although it has seldom been used, angiography shows a hypovascular cyst exhibiting an enhancing rim of contrast in the wall and septa. In the management of the current patient, ultrasound, CT and angiographic examinations prior to resection were strongly suggestive of a biliary cystadenoma.

The occasional association of a biliary cystadenoma with an anomalous bile duct supports the possibility of a congenital origin for the lesions[17]. Many investigators consider biliary cystadenomas to be neoplastic because of their multilocular nature along with their dense cellular wall and opious secretion [18]. In support of congenital origin is the observation of an aberrant bile duct in a cystadenoma[10] and in a cystadenocarcinoma[11]. In the current patient, the communication of cystadenoma to the large intrahepatic bile duct, its bile-like content, and the biliary type lining of the cyst provide further evidence in support of a congenital origin. Although there have been a few tumors containing cholesterol crystals and calcifications of the septa[11,14], this is the first such tumor with cholesterol-appearing gallstones within the cyst. Associated cholelithiasis within the normally located gallbladder has been reported[17], and apparently was a previous observation in our patient.

The occasional report of malignant degeneration has led to the assumption that these tumors are premalignant. At least two cystadenocarcinomas have been found to have arisen in benign cystadenomas; one in the liver[19] and another in an extrahepatic bile duct[4]. The authors have observed a third[25]. Another case presented by Cruickshank had malignant change in mesenchymal stroma rather than in the epithelium[20]. O'Shea[4] described a biliary cystadenoma in the common bile duct which underwent malignant changes four years following local resection. The recurrence rate for benign lesions of the extrahepatic duct following local excision has been reported as high as 22% versus 5.5% after radical resection[21,22]. Lewis and associates, reviewing their experiences, concluded that complete surgical resection is the treatment of choice[23], a technique subsequently modified as enucleation of intrahepatic biliary cystadenomas[24]. Our technique consisted of resection of the liver, with shelling of part of the tumor from its hepatic bed under the guidance of intraoperative ultrasound. We believe that a significant incidence of recurrence and the potential for malignant transformation lend support to the thesis that biliary cystadenoma be considered as potentially malignant, be treated by resection rather than by local excision and with follow up of the patient for an indefinite period of time.

### *References*

1. Pelish, T.L. and Roberts, J.A. (1987) Cystadenoma of the biliary system presenting as an abdominal mass in pregnancy. *Obstetric and Gynecology*, **70**, 466–468
2. Marcial, M.A., Hauser, S.C. and Cibas, E.S. (1985) Intrahepatic biliary cystadenoma: clinical, radiological, and pathological findings. *Digestive Diseases and Sciences*, **31**, 884–888
3. Hodgson, T. and Fox, S. Extra-hepatic biliary cystadenoma in association with adenomyomatosis of the gallbladder. *Clinical Radiology*, **43**, 210–212

4. O'Shea, J.S., Shah, D., *et al.* (1987) Biliary cystadenocarcinoma of extrahepatic duct origin arising in previously benign cystadenoma. *American Journal Gastroenterology*, **82**, 1306–1310
5. Okamura, T., Murakami, S., Nishioka, Y., Hirayama, R. and Mishima, Y. (1987) Biliary cystadenoma of the extrahepatic bile ducts: report of a case and review of the literature. *Japan Journal of Surgery*, **17**, 281–288
6. Beretta, E., De Franchis, R., Staudacher, C., Faravelli, A., *et al.* (1986) Biliary cystadenoma: an uncommon cause of recurrent cholestatic jaundice. *American Journal of Gastroenterology*, **81**, 138–140
7. Wheeler, D.A. and Edmondson, H.A. (1985) Cystadenoma with mesenchymal stroma (CMS) in the liver and bile ducts. A clinicopathologic study of 17 cases, 4 with malignant change. *Cancer*, **56**, 1434–1445
8. Petters, R.L. and Craing, J.R. (1986) Chapter 18, Neoplastic diseases. In: *Liver Pathology*, 1st ed. pp 337-364. New York: Churchill Livingstone
9. Short, W.F., Nedwich, A., Levy, H.A. and Howard, J.M. (1971) Biliary cystadenoma. Report of a case and a review of the literature. *Archives of Surgery*, **102**, 78–80
10. Quenu, M.J. (1936) Ungros kyste non parasitaire du foie. *Me'm Acad. Chir.*, **62**, 1425–1426
11. Ishak, K.G., Willis, G.W., Gummins, S.D. and Bullock, A.A. (1977) Biliary cystadenoma and cystadenocarcinoma: Report of 14 cases and review of literature. *Cancer*, **39**, 322–338
12. Forrest, M.E., Cho, K.J. and Shields, J.J. (1980) Biliary cystadenomas: Sonographic-angiographic-pathologic correlations. *American Journal of Roentgenology*, **135**, 723–727
13. Williams, J.G., Newman, B.M., Sutphen, J.L., and Madison, J. (1990) Hepatobiliary cystadenoma: A rare hepatic tumor in a child. *Journal of Pediatric Surgery*, **25**, 1250–1252
14. Geist, D.C. (1955) Solitary nonparasitic cyst of liver. *Archives of Surgery*, **71**, 867–880
15. Keech, M.K. (1951) Cystadenoma of the pancreas and intrahepatic bile ducts. *Gastroenterology*, **19**, 568–574
16. March, J.L., Dahms, B. and Longmire, W.P. (1974) Cystadenoma and cystadenocarcinoma of biliary system. *Archives of Surgery*, **109**, 41–43
17. Byrne, D.J., Walker, M.A., Pringle, R. and Ramesar, K. (1989) Extrahepatic biliary cystadenoma: report of a case and review of the literature. *Journal of the Royal College of Surgery Edinburgh*, **34**, 223–224
18. Cahill, C.J., Bailey, M.E. and Smith, M.G.M. (1982) Mucinous cystadenoma of the liver. *Clinical Oncology*, **4**, 171–177
19. Woods, G.L. (1981) Biliary cystadenocarcinoma: Case report of hepatic malignancy originating in benign cystadenoma. *Cancer*, **47**, 2936–2940
20. Cruickshank, A.H., Spareshott, S.M. (1971) Malignancy in natural and experimental hepatic cysts: Experiments with aflatoxin in rats and the malignant transformation of cysts in human livers. *Journal of Pathology*, **104**, 185–190
21. Uodff, E.J., Harrington, D.P., Kaufman, S.L., *et al.* (1979) Cystadenoma of the common bile duct demonstrated by percutaneous transhepatic cholangiography. *American Journal of Surgery*, **43**, 662–664
22. Burhans, R. and Myers, R.T. (1971) Benign neoplasms f the extrahepatic biliary ducts. *American Journal of Surgery*, **30**, 161–166
23. Lewis, W.D., Jenkins, R.L., Rossi, R.L., Munson, L., *et al.* (1988) Surgical treatment of biliary cystadenoma. A report of 15 cases. *Archives of Surgery*, **123**, 563–568
24. Pinson, C.W, Munson, J.L. and Braasch, J.W. (1989) Enucleation of intrahepatic biliary cystadenomas. *Surgery, Gynecology & Obstetrics*, **168**, 535–538
25. Lei, S. and Howard, J.M. (1992) Biliary cystadenocarcinoma arising from benign cystadenoma. A letter to the editor. Accepted for Publication, *Archives of Surgery*

*(Accepted by S. Bengmark 10 March 1993)*

### INVITED COMMENTARY

The authors of this case report have done us a service by reviewing some of the literature on biliary cystadenoma, and pointing the way to rational management.

Liver cysts lack the glamour of hepatic or biliary cancer, but it is important for hepatobiliary surgeons to understand the pathology and behaviour of the non-parasitic cysts, so that they can develop rational policies for management.

Biliary cystadenoma is — to me anyway — a puzzling entity. Incidentally discovered liver cysts are reasonably common, and most are managed conservatively. Those that I have observed for many years have generally shown no change in size, nor any change in the appearance of the wall that might suggest neoplastic progression. Although it is usually said that cystadenomas are multilocular and have characteristic appearances on ultrasound or CT scanning, an unpublished study from two teaching hospitals in Sydney, Australia, demonstrated that diagnosis of cyst type by organ imaging was unreliable, and that congenital cystic disease, cystadenoma and hydatid disease could not be reliably differentiated by experienced radiologists working closely with specialised hepatobiliary units. It is evidently important to diagnose hydatid disease, but it is not clear whether it is so important to distinguish congenital simple cysts from cystadenomas, because we do not know the natural history of the cystadenoma.

There is legitimate concern about the malignant potential of cystadenomas, and in this respect isolated case reports, rather than large series, have had considerable power in shaping surgical thinking[1,2,3]. Cystadenocarcinoma and even squamous cell carcinoma have been reported as arising in cystadenomas[1–4], but their rarity can be gauged by the number of cases cited in the comprehensive review by Ishak and colleagues in 1977[4]. They found 10 cases of cystadenocarcinoma in the medical literature by that time. Malignant change must remain a concern for hepatobiliary surgeons, but its likelihood appears to be low, and would certainly not constitute an indication to operate on every asymptomatic cyst that is picked up fortuitously by ultrasound or CT scanning.

Symptomatic systs certainly need treatment[5–7]. But what constitutes adequate treatment? There is increasing evidence that percutaneous aspiration or drainage does not provide adequate control[8]. There may be temporary relief of discomfort, but recurrence is the rule for both congenital cysts and cystadenomas. Infection in cystic disease is dangerous, and has a significant mortality[6,9]. Percutaneous drainage may be useful in gaining control over sepsis, but repeated percutaneous drainage of uninfected cysts could introduce infection, converting a nuisance into a life-threatening event. Surgical removal still provides the best treatment for symptomatic cysts.

There is an additional risk in percutaneous aspiration in countries where hydatid disease is a problem. Although there has been some interest amongst radiologists in the percutaneous treatment of hydatid disease, the first death from complications of this technique has been reported[10].

What practical advice should one give for the clinician confronting a patient with a liver cyst or cysts. The answer is easy if there are symptoms, as there were in this present case. Surgical removal is to be preferred if the patient is able to undergo surgery. Small cysts and those that cause no symptoms can be watched, once hydatid disease has been excluded. Surveillance should be meticulous because carcinoma *may* develop. The risk seems to be small, but its level is not known. Annual ultrasound or CT scans should check to see whether the cyst is enlarging or its wall is becoming thicker or locally proliferative. A change that suggests neoplasia should prompt the surgeon to advise removal of the cyst.

### *References*

1. Marsh, J.L., Dahms, B., and Longmire, W.P. (1974) Cystadenoma and cystadenocarcinoma of the biliary system. *Arch. Surg.*, **109**, 41–43
2. Ishak, K.G., Willis, G.W., Cummins, S.D. and Bullock, A.A. (1977) Biliary cystadenoma and cystadenocarcinoma. Report of 14 cases and review of the literature. *Cancer,* **39**, 322–328
3. Foster, J.H. and Berman, M. (1977) Solid liver tumours, in *Major Problems in Clinical Surgery*, Vol 22, WB Saunders Co. ,Philadelphia
4. Greenwood, N. and Orr, W.M. (1972) Primary squamous cell carcinoma arising in a solitary non-parasitic cyst of the liver. *J. Pathol.*, **107**, 145–148
5. Lin, T.Y., Chen, C.C. and Wang, S.M. (1968) Treatment of nonparasitic disease of the liver: a new approach to therapy with polycystic liver. *Ann. Surg.*, **168**, 921–927
6. Wong, J. and Little, J.M. (1977) Benign non-parasitic cysts of the liver: a review of 18 cases. *Aust. NZ J. Surg.*, **47**, 209–215
7. Lai, E.C.S. and Wong, J. (1990) Symptomatic nonparasitic cysts of the liver. *World J. Surg.*, **14**, 452–456
8. Saini, S., Mueller, P.R., Ferrucci, J.T. Jr, Simeone, J.F., Wittenberg, J. and Butch, R.J. (1983) Percutaneous aspiration of hepatic cysts does not provide definitive therapy. *Am. J. Radiol.*, **141**, 559--560
9. Robson, G.B. and Fenster, F. (1964) Fatal liver abscess developing in polycystic liver. *Gastroenterology*, **47**, 82–84
10. Vishnevski, V.A., Pomelov, V.S., Gavrilin, A.V., Ikramov, R.Z. and Viliavin, M.I. (1992) First experience in treatment of hepatic echinococcal cyst by percutaneous puncture drainage. *Khirurgiia (Mosk)* **1**, 22–26. (In Russian with English abstract)

J. M. Little
Department of Surgery
Westmead Hospital
Westmead N.S.W. 2145
Australia

